# Supplementary material for: Identification of cell states using super-enhancer RNA
Source: BMC Genomics. 2021 Nov 2;22(Suppl 3):787. doi: 10.1186/s12864-021-08092-1 (PMC8564956; doi:10.1186/s12864-021-08092-1)
Supplement: Supplementary file 1 — Additional file 1: Figure S1. Definition of super-enhancer RNA. Figure S2. Heat map of the cell type correlation matrix. Figure S3. NMF decomposition of the time-coursed macrophages response to the LPS experiment. [file 12864_2021_8092_MOESM1_ESM.pdf]

Additional File 1

Identification of Cell States Using Super-Enhancer RNA

Yueh-Hua Tu, Hsueh-Fen Juan, Hsuan-Cheng Huang

Figure S1. Definition of super-enhancer RNA.

Figure S2. Heat map of the cell type correlation matrix.

Figure S3. NMF decomposition of the time-coursed macrophages response to the LPS experiment.

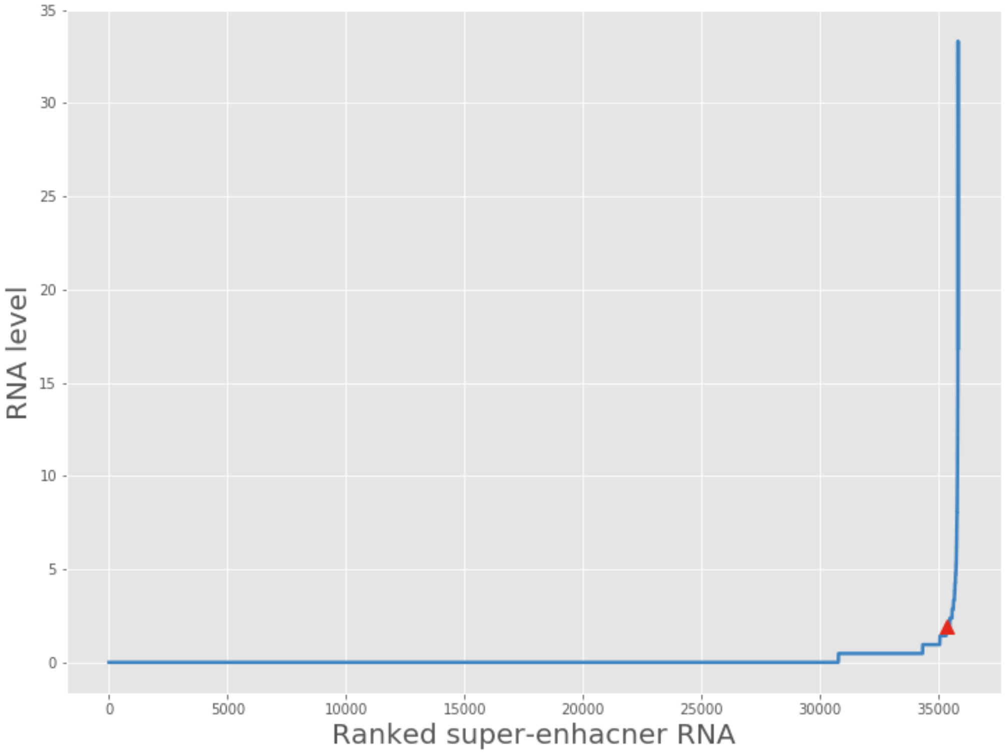

Figure S1. Definition of super-enhancer RNA. The enhancer cluster was merged by summing eRNA levels. Super-enhancer RNAs are defined as the entities to the right of the red triangle.

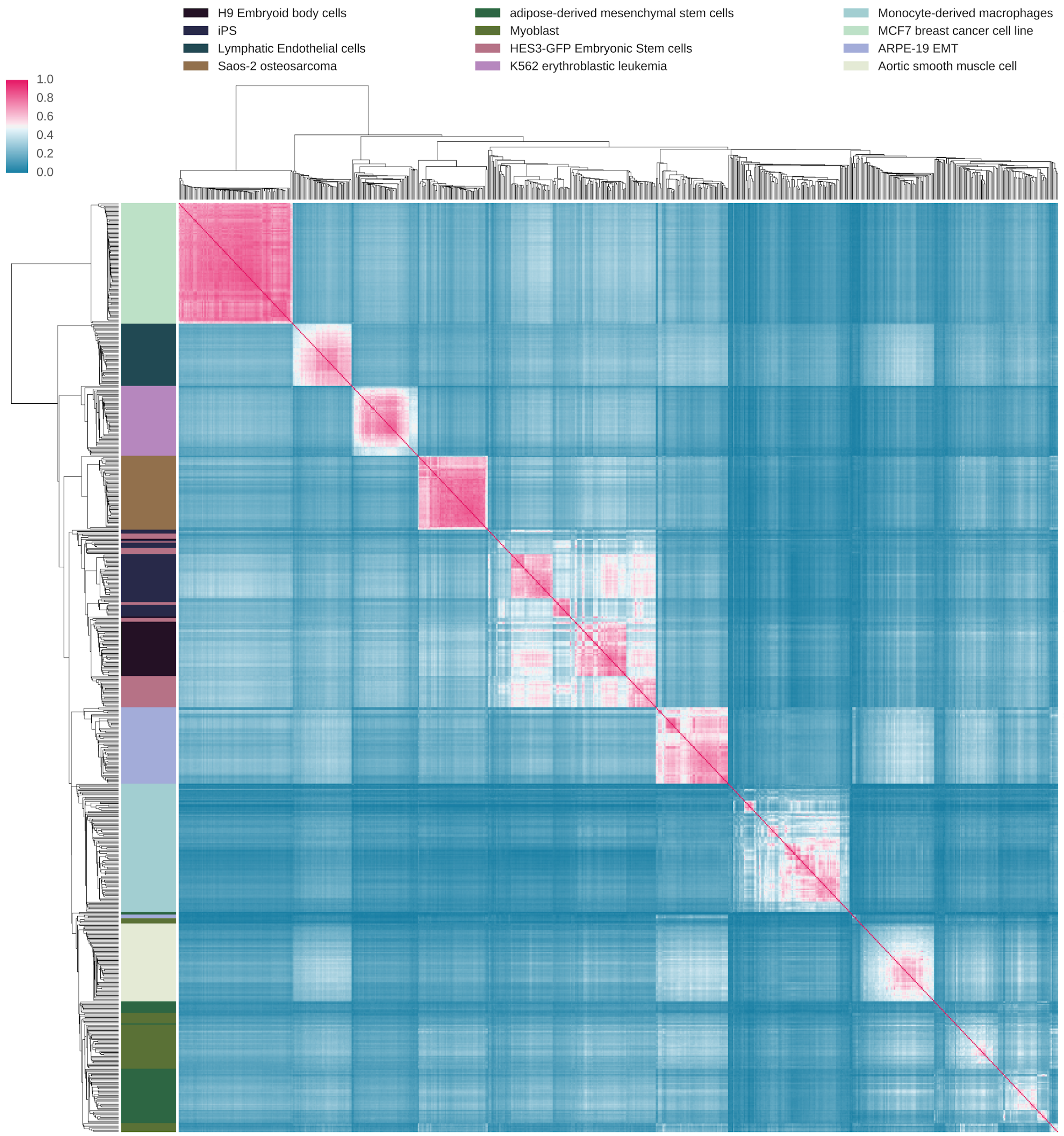

Figure S2. Heat map of the cell type correlation matrix.

Pairwise Pearson correlation coefficients were calculated among samples using super-enhancer RNA profiles. Both axes are samples with different cell types. Hierarchical clustering is performed on both axes. Cell types are clustered together, showing the similarities between super-enhancer RNA profiles from different cell types.

A

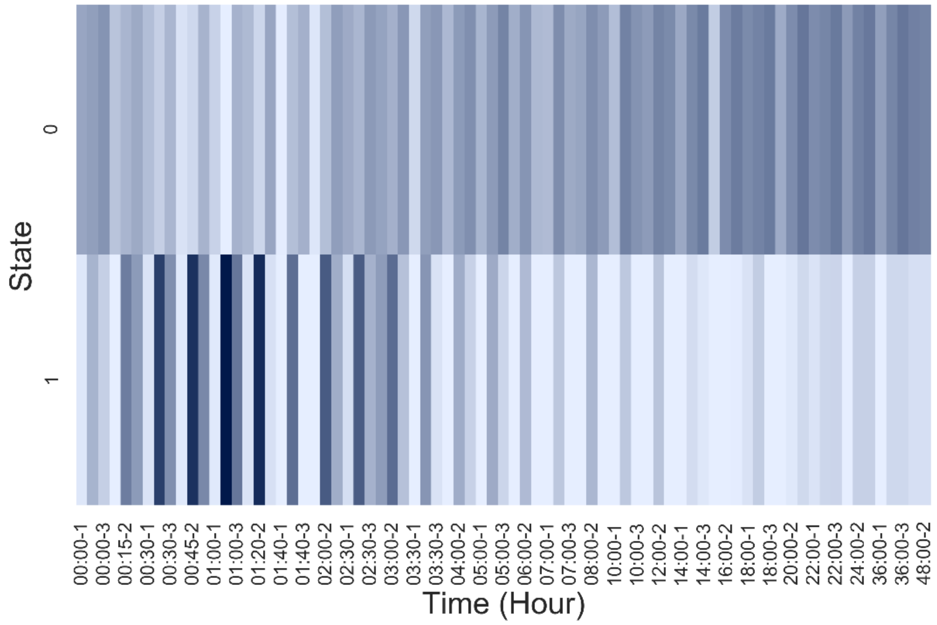
State
0
1
00:00-1
00:00-3
00:15-2
00:30-1
00:30-3
00:45-2
01:00-1
01:00-3
01:20-2
01:40-1
01:40-3
02:00-2
02:30-1
02:30-3
03:00-2
03:30-1
03:30-3
04:00-2
05:00-1
05:00-3
06:00-2
07:00-1
07:00-3
08:00-2
10:00-1
10:00-3
12:00-2
14:00-1
14:00-3
16:00-2
18:00-1
18:00-3
20:00-2
22:00-1
22:00-3
24:00-2
36:00-1
36:00-3
48:00-2
Time (Hour)

B

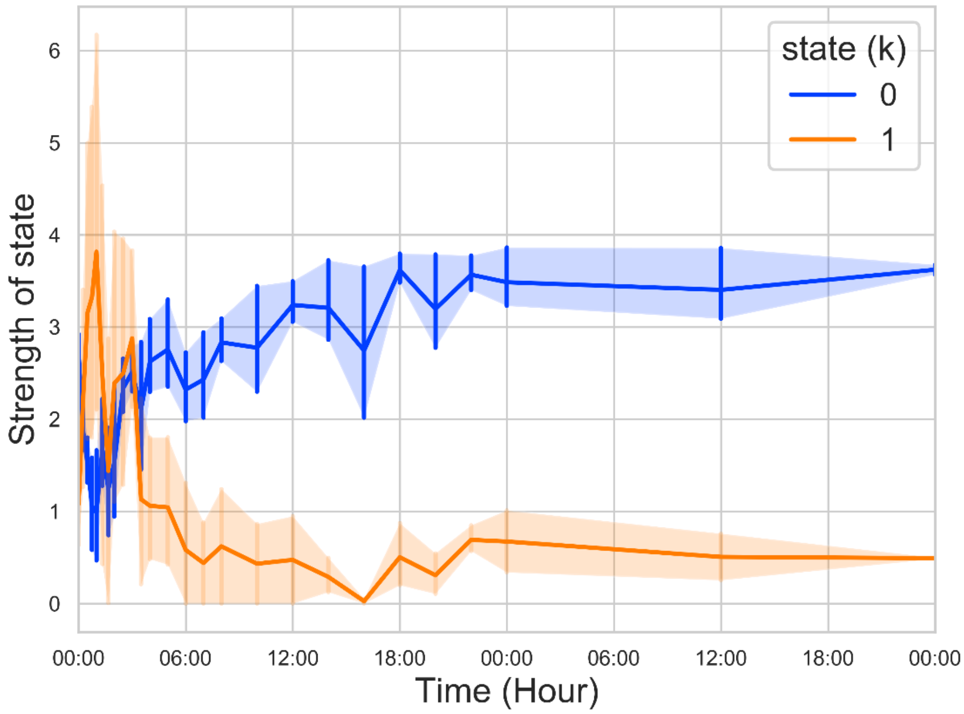
Strength of state
0
1
2
3
4
5
6
state (k)
0
1
00:00
06:00
12:00
18:00
00:00
06:00
12:00
18:00
00:00
Time (Hour)

Figure S3. NMF decomposition of the time-coursed macrophages response to the LPS experiment.

(A) State × sample matrix of decomposition from super-enhancer RNA profiles. The x-axis and y-axis stand for the time points and cell states, respectively, while the darker band represents the cell state's greater preference. (B) The time series plot was made by collapsing the biological replicates. The stimulation peak, which can be observed on both figures, was interpreted as cells that are stimulated and transition into an active state.
